# Supplementary material for: Antifungal Synergy: Mechanistic Insights into the R-1-R Peptide and Bidens pilosa Extract as Potent Therapeutics against Candida spp. through Proteomics
Source: Int J Mol Sci. 2024 Aug 16;25(16):8938. doi: 10.3390/ijms25168938 (PMC11354716; doi:10.3390/ijms25168938)
Supplement: Supplementary file 1 [file ijms-25-08938-s001.zip › Supplementary Data.pdf]

International Journal of
Molecular Sciences

MDPI

Supplementary Data

Table S1. Measurement of proteins concentration

Samples	Replica	Protein concentration (µg/µL)	Sample total volume (µL)	Total protein (µg)
C. albicans SC5314 Basal	**1**	2,38	100	238,34
	**2**	3,46	100	345,61
	**3**	3,29	100	328,93
	**4**	2,93	100	292,60
C. albicans SC5314 (R-1-R MIC-100 ug/mL)	**1**	1,66	100	166,23
	**2**	1,27	100	127,49
	**3**	1,46	100	145,97
	**4**	1,44	100	144,35
C. albicans SC5314 (Combination)	**1**	1,72	100	171,59
	**2**	1,48	100	147,76
	**3**	2,06	100	205,95
	**4**	1,19	100	118,79
C. albicans 256 Basal	**1**	5,18	100	518,44
	**2**	3,42	100	342,04
	**3**	4,52	100	452,37
	**4**	5,68	100	567,57
C. albicans 256 (R-1-R MIC-100 ug/mL)	**1**	1,35	100	134,64
	**2**	1,57	100	156,69
	**3**	1,24	100	123,92
	**4**	0,99	100	99,27

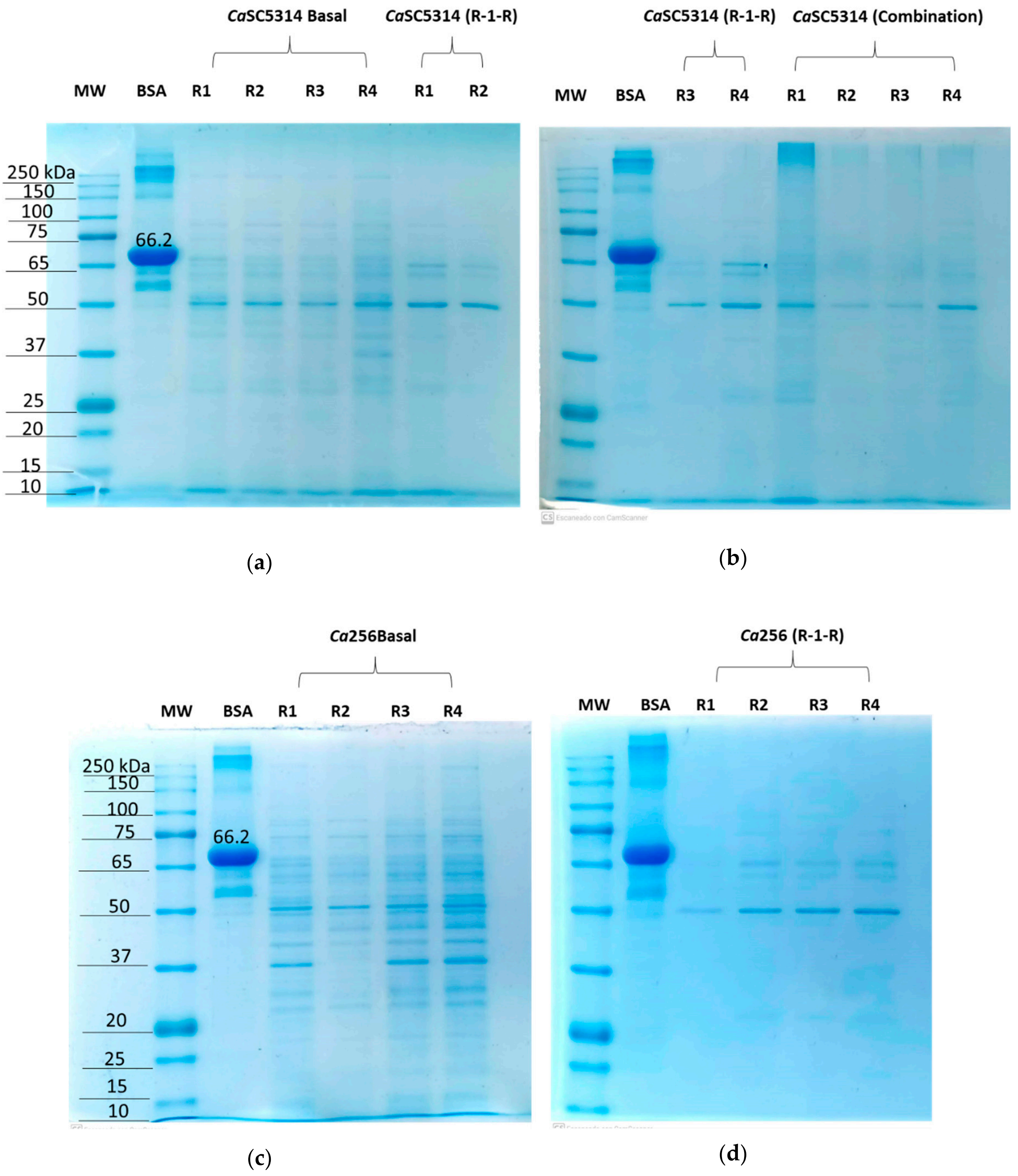

Figure. S1 The total proteins of drugs-treated (**a-b**) *C. albicans* SC5314 and (**c-d**) *C. albicans* 256 were separated by SDS-PAGE. MW: Molecular weight, BSA: bovine serum albumin, R: Replica
